# Laparoscopic guided minilaparotomy: a modified technique for management of benign large ovarian cysts

**DOI:** 10.1186/s12905-022-01853-4

**Published:** 2022-07-04

**Authors:** Mohamed F. Shaltout, Ahmed M. Maged, Rana Abdella, Mona M. Sediek, Sherif Dahab, Moutaz M. Elsherbini, Rasha O. Elkomy, Sherif Sameh Zaki

**Affiliations:** Obstetrics and Gynecology Department, Cairo University, Kasr Alainy Street, Giza, 12111 Egypt

**Keywords:** Adhesion formation, Benign large ovarian cyst, Laparoscopy, Minilaparotomy, Ovarian reserve

## Abstract

**Background:**

The aim of the study is to evaluate the efficiency and safety of a novel technique to treat large benign ovarian cysts combining benefits of laparoscopic management along with mini-laparotomy without affection of the ovarian reserve.

**Methods:**

The study included 112 women with large benign ovarian cyst candidate for ovarian cystectomy. The technique started with laparoscopy followed by guided cyst aspiration followed by exteriorization of the ovary through minilaprotomy and completion of cystectomy through microsurgical technique. The primary outcome was ipsilateral recurrence of the cyst. Other outcomes included ovarian reserve assessment and postoperative pain.

**Results:**

The number of women with recurrence in the ipsilateral ovary after 12, 18 and 24 months were 5 (4.5%),16 (14.3%),20 (17.85%) respectively. Assessment of ovarian reserve revealed a significant decrease in the level of serum AMH (2.82 ± 0.44 vs. 2.50 ± 0.42) and a significant increase in AFC (3.5 ± 1.7 vs. 4.9 ± 1.3) after our novel technique in surgical treatment of ovarian cysts (P value < 0.001). The operative time was 50 ± 7 and 62 ± 7 min in unilateral and bilateral cysts respectively.

**Conclusions:**

Laparoscopic guided minilaparotomy is a safe and effective technique for the management of large benign ovarian cysts with minimal recurrence rate, ovarian reserve affection and adhesions.

*Trial registration*: clinical trial registry no. NCT03370952*. Registered 13 December 2017,* https://clinicaltrials.gov/ct2/show/NCT03370952

## Key message

Laparoscopic guided minilaparotomy is a safe effective technique for management of large benign ovarian cysts with minimal affection of ovarian reserve.

## Introduction

The incidence of ovarian cysts is 5–15% and it shows minimal variations with different demographic data [1] yet many of these cysts are functional. The rest are neoplastic being mostly benign [2].

Whiteman and coworkers reported that benign ovarian cysts represented 7% of women admission for gynecological management [3].

Persistent simple ovarian cysts reaching 10 cm or more and complex cysts are candidate for surgical intervention [4].

The surgical treatment of benign ovarian cysts is dependent on many clinical factors [2].

Approaches include laparotomy and laparoscopy. The laparoscopic approach is preferred in cases presumed benign [4] Removing the cyst intact for pathologic analysis may mean removing the entire ovary, though a fertility sparing surgery should be attempted in younger women [4].

When compared to laparotomy, laparoscopic management has many advantages to the patient and is safe for both cystectomy and ovariectomy procedures [5, 6].

Laparoscopy offers faster recovery, better cosmesis, less pain felt postoperatively and less adhesion when compared to laparotomy [6, 7].

Removal of the ovarian cyst with preservation of the ovary can be offered to women to preserve ovarian hormonal and reproductive functions. The main points that should be considered in ovarian cystectomy is the gentle handling of tissues to minimize adhesions and to reconstruct the normal anatomy of the ovary allowing the normal ovum pick up procedure by the fallopian tube [2].

The difference between laparoscopy and laparotomy is not only the mode of access to the operative field but also non palpation of tissues, counterintuitive motion, limited tissue movements and the replacement of the three dimensional eye image by the two dimensional monitor image [2, 7].

There are traditional beliefs that laparotomy is preferred for women with large cysts and in cases of adhesions that may limit access to the cyst and its mobility [2].

The aim of our study is to keep benefits of laparoscopic management in women with large ovarian benign cysts without increasing the risks of affecting the ovarian reserve resulting from cauterization through a novel combined laparoscopic and minilaparotomy technique. The novel technique also aims to minimize pelvic adhesions, ensure complete removal of large ovarian cysts and reduce unintended gross spillage in case of an unexpected ovarian malignancy.

## Methods

A prospective cohort study was conducted at Kasr Alainy medical school, Cairo university hospital on 112 women admitted to gynecology department with the diagnosis of large ovarian cysts during the period from December 2017 to July 2019. All participants signed an informed written consent after full explanation of the procedure and potential risks and benefits. The study was approved by gynecology and obstetrics department ethical committee. The trial was registered on 13/12/2017 with NCT03370952 number.

All participants were candidate for ovarian cystectomy for a large benign ovarian cyst and their age ranged from 18 to 35 years old.

The diagnosis of benign nature of the cyst was based on clinical evaluation and confirmed by 3D ultrasound examination and Doppler studies.

Inclusion criteria included women with unilateral or bilateral ovarian cyst with a mean diameter of 10 cm or more and having a good ovarian reserve (diagnosed with antimullerian hormone > 1 ng/ml & antral follicular count > 4).Women with solid ovarian masses, those who were unfit for surgery and women with body mass index more than 30 were excluded from our study. Exclusion criteria also involved women with contraindications for laparoscopy as women with excessive anterior abdominal wall scarring and women with chronic disease as cardiac and respiratory conditions.

All participants were subjected to full history followed by complete physical examination and evaluation of ovarian reserve through measurement of AntiMullerian hormone along with the routine preoperative investigations. CA125 level was measured in all women.

Day 2 transvaginal ultrasound (or transrectal in women with intact hymen) was done using a 7.5 MHz vaginal probe of the General Electric Voluson E8 ultrasound unit (GE Healthcare Austria GmbH, Seoul, Korea) to confirm the presence and assess the size, side, consistency of the ovarian cyst & to assess the AFC (Number of visible follicles from 2 to 10 mm) in both ovaries. Transabdominal evaluation of the cysts followed using the same machine.

Under general anaesthesia, the patient is placed in the modified dorsal lithotomy position (to ensure lax anterior abdominal wall). The patient is then prepped and draped in the usual fashion for an abdominal and vaginal procedure. Whenever vaginal exam is possible, a vaginal speculum is inserted to expose the cervix; a uterine manipulator is inserted in the cervix followed by placement of a Foley’s catheter in the bladder. As regards port placement, a 10-mm umbilical trocar is used to enter the abdomen. A panoramic view of the pelvis is then obtained together with full assessment of the ovarian cyst. Any surrounding adhesions are first cut to free the wall of the cyst before aspiration to avoid blind traction on these adhesions exposing the patient to various organ injuries.

Veress needle is inserted in the midline 2 cm above the symphysis pubis to aspirate the cyst under laparoscopic guidance (to guide the entry of the needle into the cyst wall). After partial cyst aspiration, a transverse mini-laparotomy is done (about 2 to 3 cm in length) in the midline 2 cm above the symphysis pubis. A long shanks artery forceps is introduced inside the abdominal cavity to grasp the top of the aspirated cyst under laparoscopic guidance. Then, the artery forceps is pulled gently to the outside to deliver the ovary with its cyst at the mini-laparotomy skin incision outside the body followed by completion of cyst evacuation through a wide suction cannula.

**Fig. 1:**
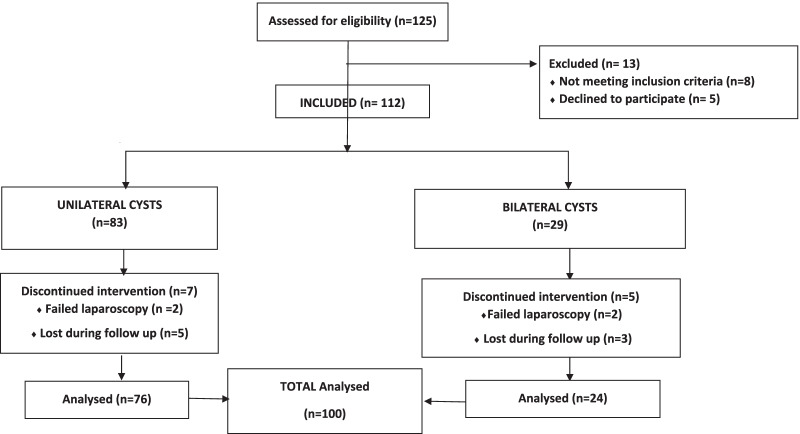
Consort flow of patients through the study

Delivering of the cyst outside the body is done only after complete evacuation of air to minimize trauma to the infundibulopelvic ligament. Careful handling and traction is applied to avoid injury of both the ovarian tissue or/and infundibulopelvic ligament. Following the delivery of the ovary, the abdominal incision is temporary closed using (E-shaped 10 × 10 cm) rubber shield (to avoid any soiling of abdominal cavity with blood or cystic fluid, particularly in case of an unexpected ovarian malignancy, and give the chance to reinflate the abdominal cavity later on) (Fig. 1).

**Fig. 2:**
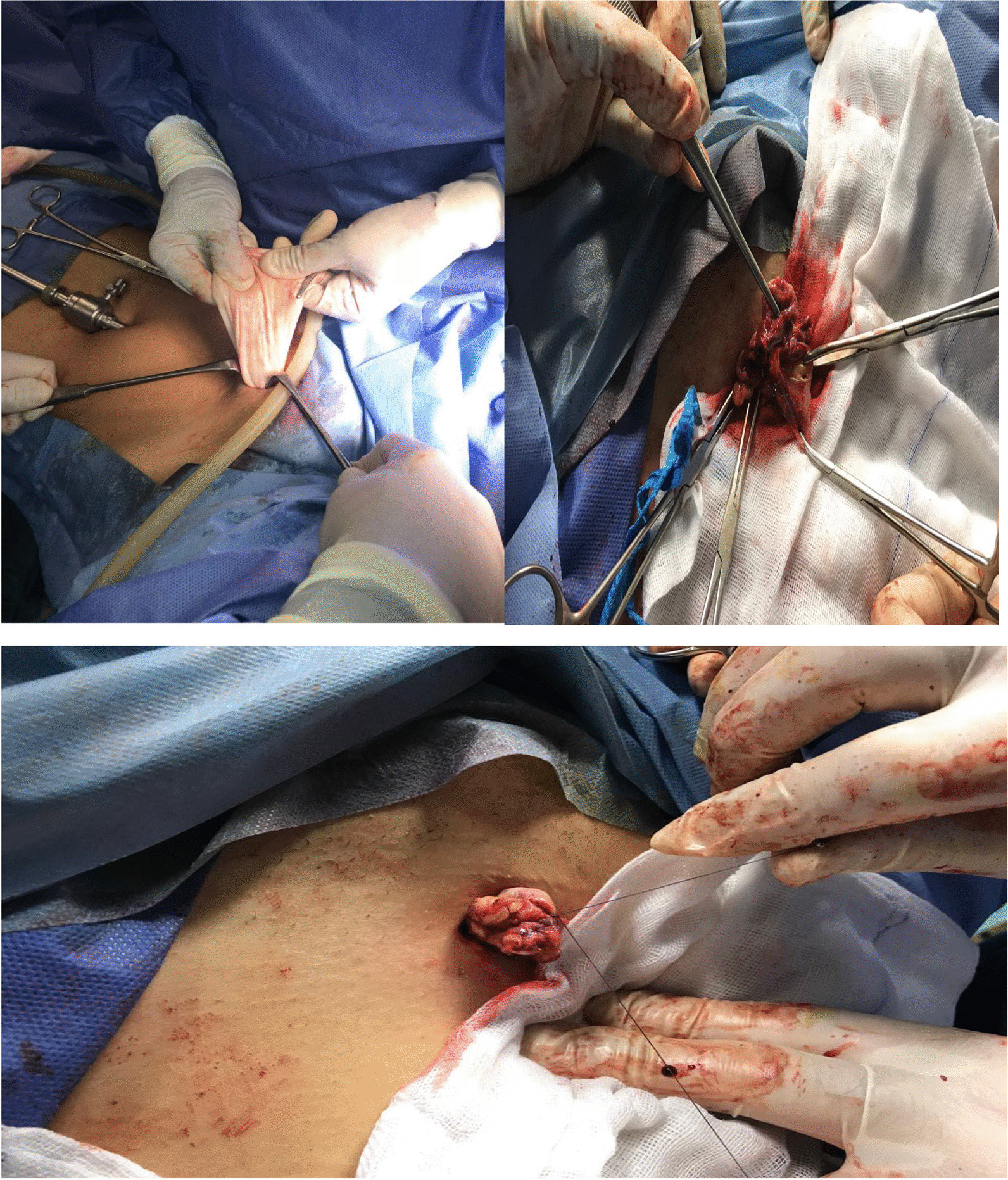
Extrusion of the cyst, Microsurgical cystectomy and ovarian closure

Classic ovarian cystectomy is done using microsurgical techniques in which the cyst wall is dissected gently and carefully from the healthy ovarian tissue followed by perfect hemostasis and re-fashioning of the remaining ovarian tissue using Vicryl 3/0 or 4/0 sutures according to the thickness of the cyst wall. Irrigation of the external ovarian surface is done using normal saline to ensure removal of any blood and minimize peritoneal contamination (Fig. 2).

The stitched ovary is pushed gently inside the abdominal cavity and the mini-laparotomy is re-covered by the rubber shield (to allow re-inflation of the abdominal cavity). The ovary is reassessed under laparoscopic guidance to ensure perfect hemostasis and normal position of the ovary. Pelvic irrigation is done using normal saline.

Closure of the abdominal incisions (trocar port & mini-laparotomy) was done.

In women with huge cysts reaching above the level of umbilicus, the operation began by the minilaparotomy incision and suction of the cyst under vision as it is seen stretching the overlying peritoneum. Suction is continued till the cyst size drops below the umbilical level. The laparoscopy is then done, grasping the top of the cyst and exteriorizing its upper part outside the body followed by completion of cyst evacuation. The rest of technique is then conducted in a similar fashion to smaller cysts.

The patient is transferred to the recovery room and discharged 12 hours later. Removal of the stitches is done after 1 week. Follow up at 2, 6, 12 and 24 months are done using ultrasonographic pelvic assessment and AMH and AFC as markers of ovarian reserve.

The primary outcome parameter was the recurrence of ovarian cysts in the ipsilateral ovary (recurrence was defined as the presence of ovarian cysts ≥ 2 cm).Other outcomes included ovarian reserve assessment, postoperative pain and patients satisfaction.

### Ethical approval

Kasr Alainy ethical committee approval number 16732 on 12/6/2016. clinical trial registry no. NCT03370952.

Statistical analysis was done using IBM© SPSS© Statistics version 23 (IBM© Corp., Armonk, NY, USA). Numerical data were expressed as mean and standard deviation or median and range as appropriate. Qualitative data were expressed as frequency and percentage. Chi-squared test was used to examine the relation between qualitative variables. A p-value < 0.05 was considered significant.

## Results

Figure 1 describe the flow chart of participants.

The characteristics of the studied population including age, gradivity, parity, body mass index and number of previous abortions are described in Table 1.

**Table 1:** Clinical characteristics of the studied group

	Description
Age (years)	27.0 ± 3.8
Gravidity	1 (0–5)
Parity	1 (0–3)
Number of previous abortion	0 (0–2)
Body mass index (kg/m^2^)	25.2 ± 1.3
Affected side	
Right	37
Left	39
Bilateral	24
Size of the cyst (cm)	15.2 ± 4.1
Cyst type	
Serous	61
Mucinous	22
Dermoid	17

Data are described as mean ± SD, median (range), or number

The size, side and nature of the ovarian cysts are shown in Table 1.

Assessment of ovarian reserve revealed a significant decrease in the level of serum AMH and a significant increase in AFC after our novel technique in surgical treatment of ovarian cysts (Table 2).

**Table 2:** Number of recurrences of ovarian masses, postoperative pain and characteristics of the postoperative period and ovarian reserve

	Description
Recurrence of ovarian masses	
After 12 months	5
After 18 months	16
After 24 months	20
VAS score of pain	
After 4 h	2 (2–4)
After 8 h	4 (2–6)
After 12 h	5 (2–7)
Shoulder pain	14
Return of Intestinal Motility	
< 6 h	23
6–10 h	63
> 10 h	14
Patient mobilization	
< 4 h	47
4–8 h	45
> 8 h	8
Anti-mullerian hormone (ng/ml)	P < 0.001
Before surgery	2.82 ± 0.44
6 months after surgery	2.50 ± 0.42
12 months after surgery	2.46 ± 0.39
24 months after surgery	2.44 ± 0.41
Ipsilateral antral follicle count	< 0.001
Before surgery	3.5 ± 1.7
6 months after surgery	4.9 ± 1.3
12 months after surgery	4.7 ± 1.2
24 months after surgery	4.6 ± 1.3
Contralateral antral follicle count	0.471
Before surgery	7.6 ± 1.8
6 months after surgery	7.5 ± 1.2
12 months after surgery	7.5 ± 1.2
24 months after surgery	7.4 ± 1.2
Total antral follicle count	< 0.001
Before surgery	11.0 ± 1.3
6 months after surgery	11.5 ± 1.0
12 months after surgery	11.3 ± 1.0
24 months after surgery	11.3 ± 1.1

Data are described as median (range), or number

The operative time is described in Table 3 and postoperative recovery including VAS pain score, intestinal motility recovery, patient mobilization and occurrence of shoulder pain is shown in Table 3.

**Table 3:** Comparison between unilateral and bilateral cases

	Unilateral Cysts n = 76	Bilateral Cysts n = 24	Total	p value
Operative time (min.)	50 ± 7	62 ± 7		< 0.001
VAS score of pain				
After 4 h	2 (2–4)	2 (2–4)		0.222
After 8 h	4 (2–6)	4 (2–5)		0.113
After 12 h	5 (2–7)	5 (3–7)		0.461
Shoulder pain	10 (13.2%)	4 (16.7%)		0.738
Return of intestinal motility				
< 6 h	23 (30.3%)	0 (0.0%)		
6–10 h	43 (56.6%)	20 (83.3%)		0.009
> 10 h	10 (13.2%)	4 (16.7%)		
Patient mobilization				
< 4 h	43 (57.9%)	3 (12.5%)		
4–8 h	29 (38.2%)	16 (66.7%)		< 0.001
> 8 h	3 (3.9%)	5 (20.8%)		
Recurrence after 24 months	15 (19.7%)	5 (20.8%)		1.000

The number of women with recurrence in the ipsilateral ovary after 12, 18 and 24 months were 5, 16, 20 respectively (Table 2).

Comparison between women with unilateral and bilateral ovarian cysts regarding the operative time, VAS pain score, occurrence of shoulder pain, intestinal recovery, women mobilization and recurrence after 24 months are shown in Table 2.

## Discussion

Laparoscopic management of ovarian cysts is dependent on many patient factors one of which is history of previous abdominal surgeries. This technique is associated with many advantages as minimal blood loss, less postoperative pain, more convenient scar appearance and shorter hospital stay. Hence the laparoscopic approach has become the standard for benign small ovarian cysts [8].

The difficulty of laparoscopic management of large ovarian tumors is related to restricted pelvic space thereby increasing the operative time and blood loss with higher possibility of conversion to laparotomy [9].

In laparoscopic ovarian cystectomy for large cysts, the rate of conversion to laparotomy was 0.45% and 2.6% and perioperative complications were reported in 1.3% and 1.6% in the Japan Society for Endoscope Surgery and Ghezzi trials respectively [10, 11].

In our experience, we observed a major issue in managing large ovarian cyst through laparoscopy, which was the increased need for cauterization of the remaining ovarian tissue to control blood loss. To our knowledge none of the investigators who used laparoscopy to manage large cysts commented on or evaluated the ovarian reserve after the procedure.

In 2004 Pelosi and colleague tried a technique of management of large ovarian cysts. They claimed that all procedures were successful without resorting to laparoscopic aid or converting to laparotomy with better cosmetic scar. They approached the cyst in 38 women through a cruciate incision where the transverse limb was performed at skin level while the vertical limb was at the anterior rectus fascia. They used a large adhesive plastic surgical wound dressing attached to the cyst surface to prevent spillage of the cyst contents into the peritoneal cavity then they aspirated the cyst till it shrinks to a size deliverable through the incision. They performed the conventional cystectomy or ovariectomy then returned the remnants to the abdominal cavity [12].

Although this technique allowed better complete removal of cyst wall without the need for the use of excessive ovarian cauterization, this apparently good technique had a major defect. The extrusion of the cyst was done blindly which expose the patient to hazards of injury of pelvic or abdominal organs as a result of traction and cutting of adhesions between the cyst and these organs blindly.

In our technique, we avoided that serious invisible injury through use of laparoscopy to locate the cyst and free it from any surrounding adhesions under laparoscopic vision before delivering it.

In our novel technique, all procedures were completed with only 4 cases out of 112 requiring conversion to laparotomy. The need for laparotomy arose from failure of laparoscopy. No operative complication were reported and most importantly the ovarian reserve evaluated after 6 months of the procedure seemed preserved. Although we observed a statistically significant decrease in the level of AMH (from 2.82 ± 0.44 to 2.50 ± 0.42 which has a minimal clinical significance), that was compensated with a significant increase in AFC. This minimal affection of the ovarian reserve was related to the use of microsurgical technique avoiding both diathermy and cold knife dissection since both affect the normal ovarian tissue adjacent to cyst wall.

Also exteriorization of the cyst allowed reconstruction of the stretched ovarian tissue over large cyst (which is not always feasible in laparoscopic surgery). This clearly allowed preservation of most of the ovarian tissue hence minimal affection of the ovarian reserve.

Our technique was successful in both unilateral and bilateral ovarian cysts. None of the operated women proved to have malignancy after pathological examination as we have strict selection criteria and proper preoperative evaluation.

Had there been an Alexis retractor available to use, we may have considered including patients with cysts suspicious of malignancy (bear in mind that large cysts are more likely to be malignant which was a challenging limitation in patient selection). The Alexis O protector-retractor has been shown to reduce scar pain, blood loss and surgical site infections or affection by malignant spillage (a common site for metastasis). It provides 360 degrees of atraumatic, circumferential retraction and protection [13].

Also we expect that our novel technique is better than both laparoscopy and laparotomy in minimizing pelvic adhesions. As a matter of fact, all risk factors of adhesions were avoided namely peritoneal trauma and exposure, avoidance of multiple ovarian tissue trauma associated with laparoscopy and towel trauma associated with laparotomy as well as the avoidance of inevitable contamination expected with laparoscopy in cysts with irritant contents as dermoid and mucinous cysts.

The main limitation of our study was the inability to confirm minimal adhesions as it needs a second look laparoscopy to assess and that wasn’t convenient to most of our patients. The reasons we believe pelvic postoperative adhesions are less with our technique would be lesser peritoneal trauma and exposure, absent peritoneal contamination with cyst contents or blood and perfect closure of the cyst by microsurgical techniques with no suture knots at the external surface of the cyst.

Another clear advantage of our technique is the assurance of complete cyst removal which can never be confirmed at laparoscopy and that eventually reflected on the low recurrence rate reported by our patients.

Machida and colleagues compared the results of laparoscopic surgery in women with different size of the ovarian cysts. They classified women to A, B and C group with cyst diameter of 5, 6–9 and more than 10 cm respectively. They concluded that the operative time and blood loss was positively correlated with cyst size. No such correlation was found between occurrence of intra or postoperative complications or conversion to laparotomy and size of the cyst. However this study didn’t comment of the use of cauterization of the ovary and the effects on ovarian reserve [9].

In a small trial involving only 12 women with large ovarian cysts, Roda and colleagues concluded that large sized ovarian cyst is not an absolute contraindication for laparoscopic management [14].

Another study was conducted by Alobaid on only 5 huge ovarian cysts managed through laparoscopy and found that laparoscopy can be used to manage huge cysts after proper patient selection with the availability of expert gynecologic endoscopy surgeon [15].

Panici et al. randomized 60 women with non-endometriotic ovarian cysts with 7 to 18 cm diameter to either laparoscopy or laparoscopically guided minilaparotomy. They found the intraperitoneal spillage was decreased with mild increase in recovery time and patient discomfort in those who underwent laparoscopically-guided minilaparotomy. And concluded that laparotomy is the standard treatment as there is lack of information about effects of peritoneal spillage. We believed that the increased recovery and discomfort was related to their large skin incision 3–7 cm compared to our 3 cm incision. The inclusion of 7–10 cm sized cysts was not appropriate as not considered as large cysts. Again they didn’t comment on the ovarian reserve of the managed women in either group [16].

Chong and colleagues compared the results of single-port assisted extracorporeal cystectomy, laparoscopy and laparotomy in 25, 33 and 25 patients with ovarian cysts. They claimed comparable outcomes with less abdominal spillage in the first procedure. However their study was a retrospective one with inclusion of cysts of 8 cm or more [17].

To the best of our knowledge, our study is unique and evaluated a novel technique and could be a standardized technique for management of large benign ovarian cysts. The limitation of that technique is the presence of dense adhesions surrounding the cyst making its laparoscopic dissection hazardous and that was not encountered in our study as a result of proper patient’s selection for the technique. The main limitation of our study was the absence of a control group with classic management of large ovarian cysts with laparotomy. We believed that all patients deserved to benefit from our technique.

The future of that technique is to apply it on women with endometriosis as they are usually young and seeking fertility and laparoscopic management with excessive cauterization could affect their ovarian reserve. We fear though, the dense adhesions normally encountered with endometriosis can pose a difficulty in exteriorizing the ovary or cause intraperitoneal opening of the cyst and subsequent spillage during attempts of laparoscopic adhesiolysis. The results need yet to be studied before such declaration can be expressed with certainty.

We can conclude that Laparoscopic guided minilaparotomy is a safe effective technique for management of large benign ovarian cysts with minimal recurrence rate, ovarian reserve affection and adhesions.

We recommend this novel technique for all women with large benign ovarian cysts who want to preserve their fertility for future fertility. Also this technique can be used in older women with confirmed benign nature of the cyst and not candidate for hysterectomy as it has a better postoperative recovery than laparotomy as confirmed by our results. We aspire to furtherly extend our study by investigating the hypothesis of lesser adhesions using this technique by performing a second look laparoscopy for that matter.

## Data Availability

The datasets generated and/or analyzed during the current study are not publicly available as the authors believe that better to be done after acceptance of the manuscript but are available from the corresponding author on reasonable request.

## Abbreviations

AFC: Antral follicular count
AMH: Anti-Mullerian hormone
VAS: Visual analogue scale
